# An optimized Arabic cyberbullying detection approach based on genetic algorithms

**DOI:** 10.1038/s41598-025-23586-8

**Published:** 2025-11-04

**Authors:** Aya M. Eissa, Shawkat K. Guirguis, Magda M. Madbouly

**Affiliations:** 1 Department of IT, Institute of Graduate Studies and Research, Alexandria University, Alexandria, Egypt; 2 Faculty of Computer and Data Science, Alexandria University, Alexandria, Egypt

**Keywords:** Arabic cyberbullying detection, Feature selection, Genetic algorithm, Mathematics and computing, Psychology, Psychology

## Abstract

The rise of cyberbullying in digital communication platforms has triggered widespread concern, not just for its reach but for the lasting psychological harm caused. Identifying such harmful behavior online is difficult in general, but when the target language is Arabic, the task becomes more complicated. The issue is not just that Arabic is written in multiple dialects, each with its own informal vocabulary, spelling variations, and structure. What complicates matters further is that meaning often shifts based on region, tone, and the social context, making abusive content harder to catch using conventional tools. This study aims to improve Arabic cyberbullying detection mechanisms by introducing a feature-selection strategy. Its main contribution involves utilizing a Genetic Algorithm (GA)-based feature selector to pinpoint harmful language patterns in a corpus of 46k Arabic Instagram comments. The GA effectively reduced the feature space by approximately half, preserving essential semantic structures while removing noise and redundancy. Four classifiers were evaluated, and GA-driven selection improved F1-scores by (3.45–14.96%) and reduced classification time by a factor of 2.32–12. These findings suggest that genetic-feature optimization enhances model precision while significantly improving runtime and reducing complexity, thereby enabling scalable, context-sensitive cyberbullying detection for Arabic and morphologically rich languages.

## Introduction

The continuous advancement of digital communication platforms has fundamentally transformed interpersonal interactions. Social media networks, instant messaging applications, and online forums have provided rapid and global modes of communication. However, these same platforms have inadvertently introduced new avenues of psychological harm, most notably in the form of cyberbullying^1–3^. Unlike traditional bullying, which tends to be physical and visible, online abuse can spiral out of control in seconds. Many of those affected report lasting symptoms such as anxiety, emotional stress, social withdrawal, and even signs of depression or, in severe cases, physical aggression, especially among teenagers. This increasing issue brings about the necessity for intelligent, dynamic tools to match the ever-changing nature of Internet communication. It has been reported that there is a necessity for developing an automated approach in detecting cyberbullying so it would not consume a lot of time and effort^4^. Nevertheless, the performance of these models is primarily a function of the choice of features that can identify abusive or offending language. When it comes to Arabic, however, the challenge gets even more complicated. The language itself is structurally rich and diverse not only because of its complex grammar and right-to-left script but also dialectal variations that add to the problem. Arabic has regional dialects that differ in grammar, vocabulary, and orthography because of contact with the English, French, Turkish, and other languages. These variations alter the context and the perception of the text, making it hard to develop a model that will accurately identify abusive or toxic language^5–8^. Compared to English, which has enough material for cyberbullying analysis, there are very few linguistic databases or vocabularies for Arabic to measure cyberbullying-related words. Similar challenges are reported in other morphologically rich, low-resource languages, such as Kurdish, where transformer-based models have been developed to handle scarcity of annotated corpora^9^. Given these challenges, feature selection becomes a crucial step in building robust classification systems for Arabic cyberbullying detection. These algorithms, inspired by the mechanics of natural evolution, are incredibly useful for navigating large, messy feature spaces. By helping reduce irrelevant data without losing the important features, GAs can sharpen machine learning models and make them better at picking up subtle linguistic or cultural cues^10–13^.

In this study, GA-based feature selection (GA-FS) was applied to improve Arabic cyberbullying detection. The idea is to combine the adaptability of GAs with robust machine learning classifiers to overcome the hurdles posed by the language’s complexity.

Our work sets out to bridge a notable gap in the field. While much of the focus in cyberbullying research is on English or other widely spoken languages, Arabic remains underserved. By proposing a culturally and linguistically aware method, we aim to move the field forward and offer a tool that actually works for Arabic-speaking communities^5–7^.

The remainder of this paper is organized as follows: Sect. “Literature review” reviews the literature of cyberbullying and detection methods in general, and specifically in the Arabic context, along with background on feature engineering, concluding with a related work summary. Section “Results” contrasts the proposed model and presents experimental results. Section “Discussion” discusses the results, implications, and recommendations for future research. Section “Conclusion” gives concluding observations.

Section “Methods” provides an outline of the suggested GA-FS model, detailing each segment of the pipeline.

## Literature review

This section introduces the basic concepts of cyberbullying and demonstrates the constructed GA-FS framework. It covers some basic concepts in feature engineering and dimensionality reduction, and then metaheuristic algorithms with emphasis on GA. Finally, it touches on recent research developments in sentiment analysis. Table 1, shown at the end of the section, offers a snapshot of the seminal and recent literature referenced in this review, classified by topic and summarizing their key contribution directly relevant to our method.

**Table 1:** Thematic summary of related works and their core insights across cyberbullying detection and feature selection.

Main Factor		Main Insight		References
Cyberbullying & Its consequences		Cyberbullying inflicts lasting harm from heightened anxiety and depression to dips in grades and is often underreported where stigma prevails.		^14,15,19,48^
Why GA?		GAs excels at sifting through massive, dialect-rich text spaces; fused with classifiers, they deliver faster, more accurate detection for Arabic.		^20–22^
Benefits of Feature Selection		Pre-filtering with hybrid or online feature selection methods cuts noise, curbs overfitting, and yields transparent feature sets boosting speed and trust.		^24–26,29^
Impacts on Models		Trimming features prevents overfitting, reduces false positives/negatives, and scales classifiers to millions of messages without data-volume collapse.		^28,30,33^
Effects on Text Classification		Tackling text sparsity especially in Arabic by spotlighting high-impact words and down-weighting noise allows SVMs and deep nets to predict reliably.		^31,32^
Metaheuristic Enhancements		Bio-inspired searches, from genetic crossover to ant-colony pheromones, uncover subtle, nonlinear text patterns, underpinning hybrid feature selection pipelines.		^34,35^
GA Specific Advances		From early GA-FS trials to modern fast approximations, improvements in encoding, crossover, and mutation converge quickly on informative feature subsets.		^38–41^

### Cyberbullying and its effects

Cyberbullying is the act of harming or harassing another person using technology or the internet, and its psychological implications are severe. The impact of cyberbullying leads to increased rates of anxiety, depression, and low self-esteem, and in extreme cases, even suicidal ideation^2,14^. Hence, while personal insults and threats might be manageable in several cases, the uniqueness of the internet as an environment that is both completely anonymous and incredibly sprawling makes cyberbullying a particularly dangerous undertaking. To the students and teenagers who are among the most active users of the Internet, cyberbullying interferes with their academic achievements, communication with peers, and mental health^15–17^.

Research on Arabic cyberbullying faces methodological difficulties because of dialectal fragmentation and limited dataset availability^5^. The social expectation of preserving family dignity makes it hard for victims to disclose their experiences. People who decide to seek help from available support services tend to use them at very low rates according to^18^. Cyberbullying remains poorly understood in most Arab regions, since people lack knowledge about how to identify or report this issue^19^.

### The necessity of applying GA to feature selection

Considering what has been discussed and the negative consequences rampantly associated with cyberbullying, it becomes important to establish a proper model for analyzing abusive language in Arabic. One of the most popular techniques is GA, that, based on the idea of natural selection, is considered to be a promising method for feature selection^20^. GA is used to sort through the features of a data set, which can include keywords, phrases, or stylistic features, and optimize machine learning for maximal accuracy^21,22^. If the same approach is applied to Arabic, then it might provide a strong solution against different dialects of the language when it comes to the identification of cyberbullying^21^.

### Pros of using feature selection before classification

This is one of the most important preprocessing steps in machine learning, where the most important features from a given data set are selected and the rest are discarded. This step is very important, especially in text classification, because datasets in text classification contain many features; for example, words, phrases, or n-grams that may overwhelm the machine learning algorithms. Feature selection, when used to select only important features, is very useful in improving the performance of the classification models as well as increasing efficiency^23–25^.

The first advantage of feature selection is the ability to cut down on the number of features, otherwise known as dimensionality reduction^26^. Due to the large variability of the texts and languages used in the creation of text classification datasets, they can contain thousands or even millions of data features. Most of these features can either be irrelevant or duplicate, due to which it might be difficult to classify. Feature selection eliminates such features on the dataset, as shown above, which may not be relevant to the solution of the problem hence bending the model to be easier and computationally inexpensive.

This issue is especially acute in processing morphologically rich languages such as Arabic, where feature selection can make a critical difference^27^. Useless or unproductive attributes distort the data in which machine learning algorithms work and worsen it by adding noise. It selects the most relevant features and helps the model ignore the rest, making it easy to classify outcomes that are most important for making a decision^28^. The effectiveness of such an improvement is especially notable in text classification problems, where even the nuances of the use of words have a critical impact. Feature selection also helps in increasing the interpretability of models in machine learning. Sometimes, it is preferable to train a model on a subset of features; the process helps decipher the reasons behind some of the outputs made. For example, in a text classification task to detect cyberbullying, the features that were selected may be centered on some special abusive words or phrases^29^. This interpretability may be useful for researchers and practitioners, who must explain the result or adjust the models.

Feature selection can help the model and prediction processes to be completed much faster. In this case, the use of machine learning algorithms is normally accompanied by significant computational resources in solving the problems. By decreasing the number of features, feature selection accelerates the construct and allows making predictions in real or near real-time, which is especially important for applications such as spam filtering, sentiment analysis, or cyberbullying identification^25^.

### Impacts of feature selection on classification models

The process of feature selection determines which attributes lead to the best generalization performance of models while minimizing overfitting effects and refining decision boundary definitions^30^. The process of selecting features plays a vital role in text classification because sparse, high-dimensional vocabularies would otherwise defeat algorithms. Feature selection enhances model interpretability by demonstrating which specific words and phrases lead predictions, which become crucial for detecting cyberbullying^29^. Feature selection reduces feature sets to improve computational speed and deliver fast predictions across extensive text datasets^25^.

### Effects of feature selection on text classification

When it comes to text classification, feature selection has a very straightforward impact on how well a model comprehends or even classifies text data^31^. This is helpful because it draws attention to those parts of the text that are likely to be informative of the target classes, the keywords, phrases, or patterns^31,32^. For instance, in cyberbullying data set, feature selection might consider words such as ‘happy’, ‘angry’, or ‘sad’, therefore excluding such words as ‘the’, ‘and,’ etc. Feature selection also offers a critical contribution to handling the problem of sparsity in text data^31^. It is worth noting that in most text datasets, there are a large number of features in which the number of occurrences is very low. In addition, feature selection makes the proportional distribution of data more balanced and representative because only those features that are most frequently used are selected. Feature selection enables the use of more sophisticated algorithms for text classification^32^. With reduced dimensionality, models such as support vector machines (SVM), decision trees, or even deep learning architectures can be implemented more effectively, resulting in better predictions.

### How metaheuristic algorithms improve feature selection for text classification models

Metaheuristic algorithms are complex optimization methods based on natural observation, which include evolution, swarm, or physical process^33^. These algorithms have been used in solving various problems in different disciplines, and they have been used in feature selection for text classification. Text categorization entails sorting text data into already defined classes, including spam or not spam, positive or negative sentiment, or the presence of cyberbullying in the text data^34,35^. Feature selection is important at this time to select the most informative and important features from the dataset while working with a smaller set of features. In this study, Metaheuristic algorithms like GA provide several benefits that enrich the aspect of feature selection as well as the text classification models at large.

### Dealing with text data complexity

The text datasets are inherently challenging to analyze because of the high dimensionality, sparse matrix, and diverse choice of words. The problem is that traditional feature selection methods, including filter and wrapper methods, are incapable of handling such complexity levels. Metaheuristic algorithms, in contrast, can be applied to any problem that has a large search space^33^. They employ the mechanism of intelligent search to find out which of the features best defines the subset and how much exploration against exploitation in the search space should be performed. For instance, techniques such as GA select solutions over several generations in a natural selection process, to identify the most important features amongst feature sets in the dataset^34,35^. Beyond GA, other nature-inspired methods, like the improved discrete laying chicken algorithm have also shown notable success in enhancing text classification performance^36^.

### Increase in the efficiency of text classification

Perhaps one of the most valuable aspects of metaheuristic algorithms in feature selection is the enhanced accuracy of text classification models^23^. These algorithms help to minimize noise and inconsequential data, which may complicate the classification models within a given dataset. In a sentiment analysis task, a metaheuristic algorithm can prefer sentiment words such as ‘happy,’ ‘angry,’ or ‘sad,’ and demote stop words such as ‘the’ or ‘and.’ These non-redundant features also assist in the accuracy of the classification model, as it provides a more sophisticated feature subset.

### Handling nonlinear relationships between features

In text classification, some features can have a complex relationship, where two or more features may interact in a non-linear manner that a basic linear model cannot pick^37^. Among these variables, metaheuristic algorithms offer an excellent fit for modeling these relationships. Particle Swarm Optimization (PSO) mimics the movement of a swarm of particles, and each particle depicts a prospective feature set. While particles are exchanging messages containing information, the algorithm finds a correlation that would give the best features, even if the relationships are not linear^37^. This capacity allows for the identification of nonlinear interactions and, therefore, improves the quality of the chosen features and, thereby, the classification model.

### Robustness to diverse text challenges

Text classification generally has several issues that include writing styles, languages, and noise within the data set. Metaheuristic algorithms are resistant to those challenges because they do not assume a lot about data^11^. Like the cooling process of metals, the simulated annealing approach can get out of the local optimum and search even for the global optimal feature subset. This adaptability makes sure that the selected features are useful throughout different text datasets, which makes the classification model generalizable^28^.

### Enhancing model interpretability

Such metaheuristic algorithms do increase the efficiency of text classification models’ performance and make those algorithms easier to interpret. These algorithms tend to find subsets of features that are smaller and more significant, to explain why a model reaches such a decision. In a cyberbullying detection task, some of the features selected might be the specific abusive words or phrases that were used, and this makes the decision quite unambiguous^29^. This is particularly important in creating trust with machine learning applications, especially within sensitive subjects such as online safety.

### Text feature selection using GA

There is another very effective technique for feature selection named GA, which is a metaheuristic optimization technique based on the principles of selection, crossover, and mutation of natural selection and evolution. In a similar way that GA emulates the ‘‘evolution’’ of solutions, it does so with feature selection, and by doing so, grants the right measure of exploration and exploitation to generate efficient and effective models^38,39^. The GA works based on a population of solutions, in which each solution consists of a population of feature subsets^40^. These are commonly called “chromosomes”, and the solutions improve through selection, crossover, and mutation to enhance feature subsets. Each of these solutions can be fit into the framework based on a set goal, for example, increasing the accuracy or precision of the text classification model. This approach is most important because it helps the algorithm to concentrate on feature subsets that are most important in the model^40^.

Since text feature selection is based on high dimensional data, GA is useful for solving this problem. Text datasets generally have thousands of, or millions of features, which are often unimportant, or which replicate other important features^33,41^. Since traditional optimization techniques fail to handle this complexity, GA is well suited to handling large search spaces. Thus, by apprehending the overall search for the best feature subsets, the dimensionality of the dataset is decreased, and the classification model becomes faster and more efficient. Recent studies, such as multi-objective manifold representation approaches, further highlight the role of optimization in capturing complex opinion patterns in text classification tasks^42^.

In feature selection, optimization methods face the problem of premature convergence to suboptimal solutions that provide low-quality feature subsets that do not represent the optimal feature subsets. GA addresses this issue by introducing genetic variation using crossover (parts of two solutions) and mutation (random changes in solutions)^39,40^. Such diversity is beneficial because it lets GA search for a wider area and identify the best feature subsets globally. As presented above, GA is a highly scalable method and is well suited to other text classification problems. In this respect, it can be used with any type of language, domain, and dataset, without any restrictions based on some assumptions made on the data^41,43^. For instance, in the case of detecting cyberbullying, GA can be tuned to identify abusive words or phrases; in the context of sentiment analysis, GA can emphasize emotional terms^21^. This flexibility renders GA a useful feature selection in virtually all areas of application.

Apart from improving the model accuracy, GA also makes text classification models easier to understand^29^. Since the model is only restricted to considering a small number of features that are most relevant in determining output, it is much easier to explain why the model makes certain predictions. For instance, the identified features in a spam detection task may point to specific words related to slim scientific interest, while unveiling the decision-making procedure of the model.

### Research gap and objectives

**Fig. 1:**
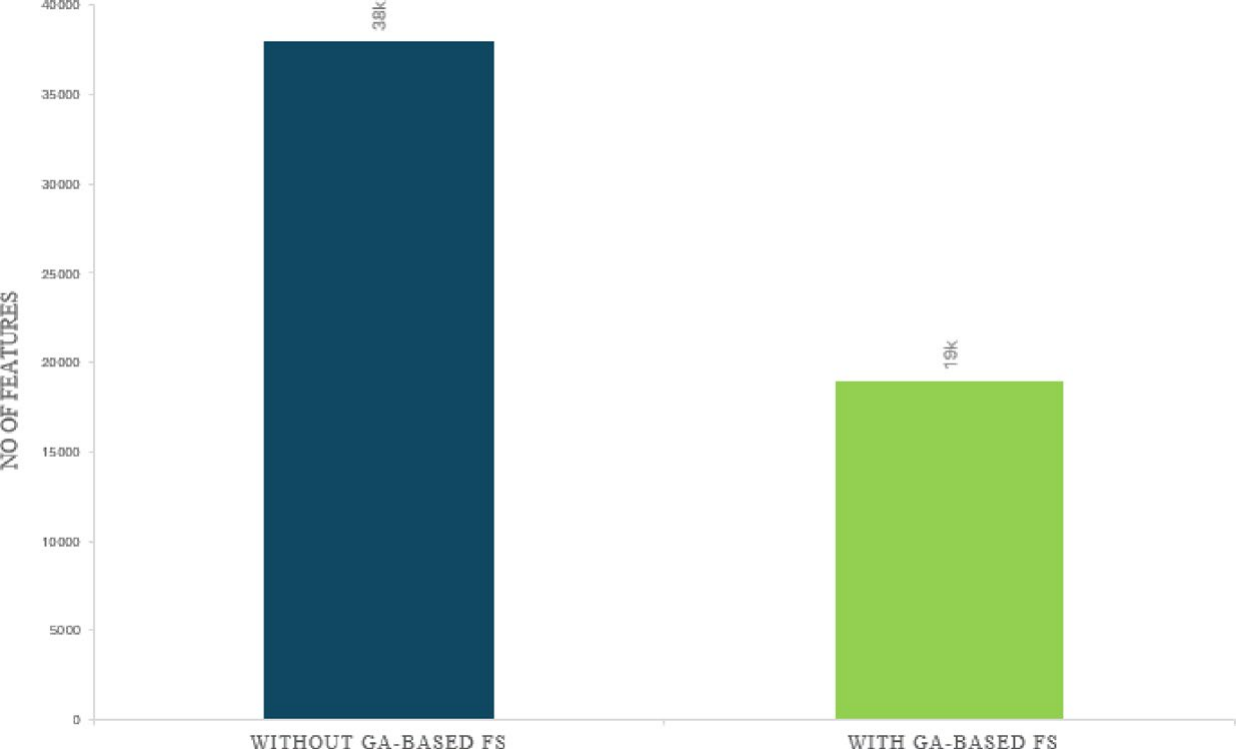
Dimensionality reduction achieved through GA-FS on the TF-IDF feature space.

Despite growing interest in Arabic cyberbullying detection, existing models struggle with high-dimensional feature spaces and lack interpretable insights into dialect-specific abuse. In this study, we apply GA–FS to distill over 38, 035 features derived from Term Frequency-Inverse Document Frequency (TF-IDF) and embeddings into a compact, highly discriminative subset (as detailed in the Results section and Fig. 1, where feature count was reduced to 19,012 using the GA pipeline described in Methods). By explicitly handling script variation, colloquial language, and cultural context, a GA-FS framework was implemented to enhance classification precision while reducing computational expense through its combination of preprocessing methods and lexical representation and GA optimization. The evaluation of feature subsets was conducted using an SVM with a weighted F1-score. The evaluation process identified the most useful features, which are employed across various classifiers for benchmarking. The selected feature set will also detect the most important abusive words, providing actionable insights for sociolinguistic examination and platform moderation.

## Results

This section reports findings in four parts: replication baseline study, impact of feature reduction, comparative performance of the four classifiers, and time-saving gains.

### Baseline models replication

Prior to assessing the impact of feature reduction, the performance of each classifier was verified against published benchmarks^44^. Using the complete set of 38, 035 language indicators, SVM, Random Forest (RF), Logistic Regression (LR), and Multinomial Naïve Bayes (MNB) achieved accuracy, precision, recall, and F1-scores within 1% point (pp) of the original results. This close correspondence confirms that data preprocessing, model configuration, and evaluation procedures were implemented correctly.

### Proposed GA-FS outcomes

A GA was employed to select the most informative 19, 012 features, exactly 50% of the original set. Figure 1 illustrates the reduction in dimensionality. The GA-FS implemented in this study initiates with a random population of feature subsets, each represented as a binary chromosome. Through iterative evaluation using an SVM-weighted F1-score, the GA evolves toward an optimal feature set by applying selection, crossover, and mutation operations, retaining features that most improve classification and eliminating those that are redundant or irrelevant. Full technical details and reasoning appear in the Literature Review and Methods. The retained features represent those with the greatest contribution to model discrimination, while redundant or low-value indicators were excluded. Despite halving the feature count, no deterioration in classifier performance was observed; in several cases, metrics improved. Even though it reduced the number of features by half, no performance degradation was found; in some cases, measurements improved.

### Classifier performance before vs. After reduction

Test set results for each model, illustrating both accuracy and balanced performance (F1-score) before and after feature reduction, are summarized in Tables 2 and 3.

**Table 2:** Evaluation metrics before using GA-FS.

Classifier	Accuracy	Precision	Recall	F1-score	Classification Time (sec)
**SVM – Before GA-FS**	69%	67%	69%	66%	11.6
**RF – Before GA-FS**	67%	65%	67%	65%	29.4
**LR – Before GA-FS**	66%	66%	59%	59%	9.7
**MNB – Before GA-FS**	66%	66%	66%	65%	0.8

**Table 3:** Evaluation metrics after using GA-FS.

Classifier	Accuracy	Precision	Recall	F1-score	Classification Time (sec)
**SVM – After GA-FS**	71.76%	71.76%	71%	71.61%	5.0
**RF – After GA-FS**	69%	69.72%	69%	68.61%	2.44
**LR – After GA-FS**	68.68%	69.85%	68.68%	67.83%	2.66
**MNB – After GA-FS**	68%	68.92%	67.82%	67.24%	0.16

- SVM: Accuracy rose by 2.76 pp and F1-score by 5.61 pp, indicating more consistent discrimination across cyberbullying detection categories when focusing on the strongest language cues.
- RF: Accuracy rose by 2 pp and F1-score by 3.61 pp, demonstrating that simplified input improved the stability of ensemble decision rules.
- LR: F1-score improved by 8.83 pp (from 59.0% to 67.83%), reflecting substantially better balance between precision and recall, particularly for the underrepresented neutral class, which often requires context to confirm bullying intent.
- MNB: Improved F1-score by 2.24 pp, so that even count-based models are enhanced by removing non-informative features.

### Computational efficiency improvements

**Fig. 2:**
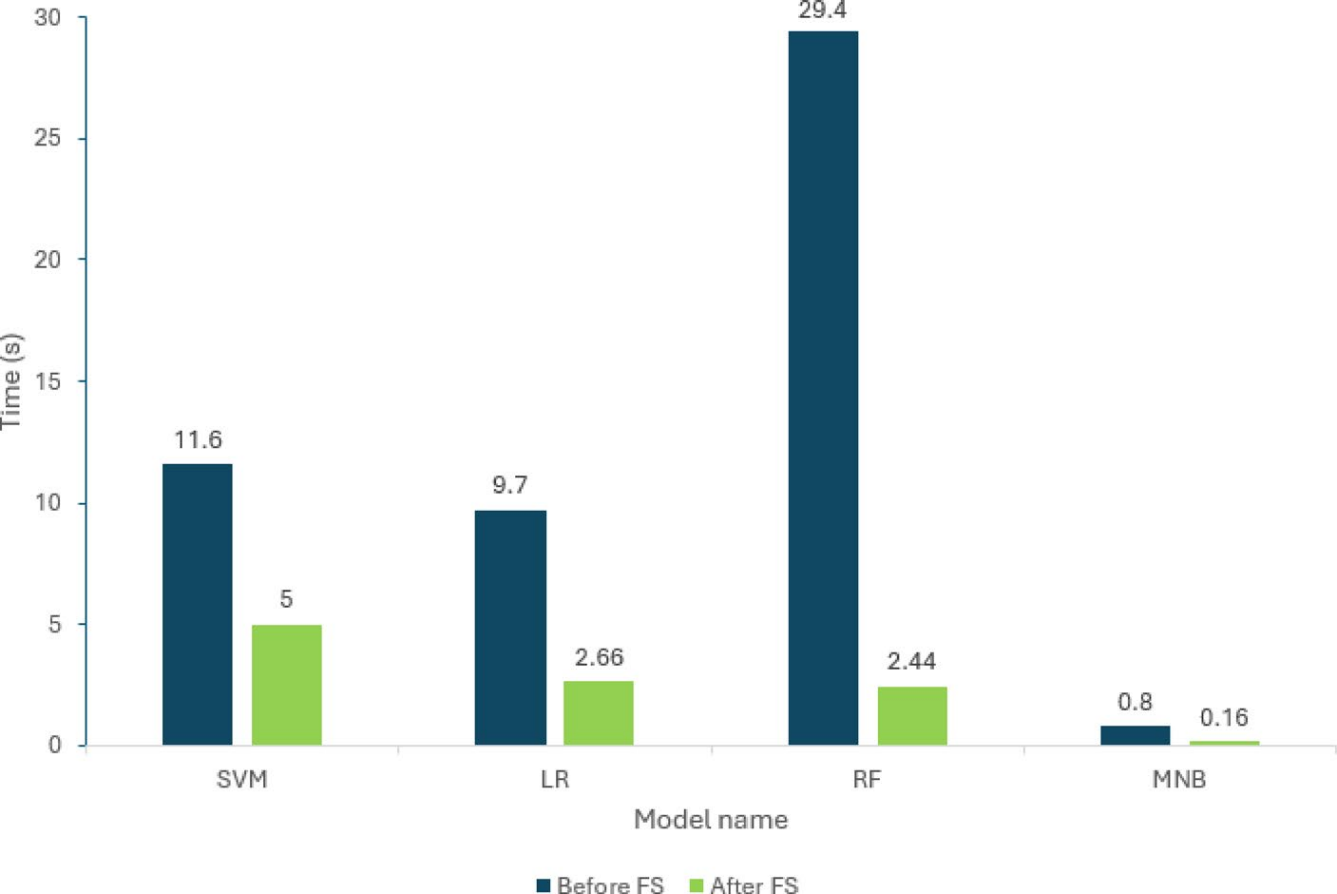
Comparison of model training and inference times before and after GA-FS.

Figure 2 shows the reduction in processing time for model training and inference. Feature reduction yielded the following improvements:

- SVM: Inference time decreased from 11.60 s to 5.00 s (57% reduction).
- RF: Inference time decreased from 29.40 s to 2.44 s (92% reduction).
- LR and MNB: Both classifiers completed test set evaluation in under 3 s, compared with approximately 10 s when using the full feature set.

These efficiency improvements allow quasi or real-time analysis in applied psychology environments to be scalable.

### Summary of findings

The methodology’s replication validated the experimental setup. GA–based feature reduction reduced dimensionality by 50% without degrading and in most cases improving classifier performance. All four models demonstrated higher accuracy and F1-scores alongside substantial reductions in inference time. Statistical testing confirmed the reliability of these improvements. These findings underscore the value of selective feature pruning for enhancing both analytic precision and operational efficiency in cyberbullying detection tasks within psychological research.

## Discussion

The present study evaluated the efficacy of GA-FS for Arabic language sentiment classification by constructing a pipeline that employs TF–IDF feature extraction. A GA is then applied to iteratively reduce the feature space by 50% (from 38,035 to 19,012 dimensions) using an SVM-based weighted F1-score as a fitness criterion. The GA process measures the predictive strength of candidate feature subsets while eliminating features that fail to enhance classification outcomes. Four supervised classifiers evaluated the compact feature set, which includes positive (not cyberbullying), negative (cyberbullying/toxic), and neutral (uncertain) cyberbullying labels. Comprehensive methodological steps and parameter settings are detailed in the Methods section for reproducibility. The succeeding paragraphs explain these results, address implications, limitations, and propose directions for future research.

LR and SVM benefited the most from feature reduction. The accuracy of SVM increased by 2.76 pp and F1-score by 5.61 pp, while that of LR increased by 8.83 pp. This increment signifies that the reduced, lower-dimensional feature set strengthened the hyperplane and linear boundary decision, respectively, by selecting the most discriminative lexical features. Noise introduced by low-value features was substantially eliminated, leading to stronger differentiation between cyberbullying categories.

RF also demonstrated performance improvements, accuracy improved by 2 pp and F1-score improved by 3.61 pp, although it is naturally resistant to high dimensional data. The lower feature set enabled each tree to identify stronger splitting features early in the ensemble process, thereby reducing variance in tree predictions. This effect was especially pronounced for inference runtime, as RF’s tree-based structure benefits substantially from a simpler feature space; fewer potential splits at each node directly reduce computational overhead and accelerate processing compared to other classifier types.

However, RF training time was still relatively high compared to SVM and LR, reflecting its computational requirement on numerous decision splits.

MNB demonstrated a modest F1-score increase of 2.24 pp. Given MNB’s assumption of feature independence, it is less sensitive to inter-feature correlations. The smaller improvement indicates that, while GA removed noisy features, MNB’s probabilistic framework was already robust to high dimensionality. Yet, the dramatic reduction of time taken for classification from 0.80 s to 0.16 s shows an operational benefit even when accuracy gains are small in models.

Building on these classifier-specific results, it is important to consider the linguistic characteristics of Arabic that may further explain the observed patterns. Arabic’s rich morphology, ample inflection, and dialectical variability are problematic for representations of bag-of-words. GA’s capacity for finding and grasping contextually useful n-gram patterns, like negation, common collocations, and dialect-specific terms, demonstrates its utility in distinguishing linguistically useful features. This selection by feature is likely the reason linear classifiers performed better, which depend heavily on clear, independent signals.

These findings also align with broader methodological trends in text classification research. Metaheuristic algorithms have become increasingly popular in English and other languages’ text classification studies because they select features according to an empirically optimized fitness function, typically based on the classifier’s performance. This practical advantage often results in better contextual selection than methods such as Chi-Square or Mutual Information, which assess features independent of the final task. Nevertheless, direct comparison with these methods remains a worthwhile direction for future research, in order to comprehensively position GA’s utility.

Beyond methodological contributions, the results also carry practical implications for cyberbullying detection in real-world settings. Results are of empirical significance to social media cyberbullying detection psychological research:

- Efficient Large-Scale Studies.

Reduced feature sets enable near real-time computation of thousands of comments, enabling large-scale bullying detection tracking and timely intervention in digital mental health technologies.

- Model Interpretability.

By limiting the feature space to the most informative predictors, researchers can more conveniently investigate which words or phrases impact bullying or non-bullying assignment and make qualitative examination of emotional lexicons convenient.

- Resource-Constrained Environments.

In settings where computational resources or bandwidth are scarce such as mobile applications or low resource field work leaner models perform well without excessively costly hardware.

Although GA reduced inference time, the feature selection process itself incurs a one-time computational cost. For extremely large corpora, this upfront investment may be non-trivial.

Alignment with Prior Research.

The latest studies show that GA-FS works well for Arabic cyberbullying-related text classification, especially when dealing with dialects, and morphological complexity. The classification performance improves significantly through^45^ hybrid filter GA as well as the Whale Optimization hybrid techniques of^46^ when compared to traditional methods such as TF-IDF and PCA. These approaches consistently highlight the advantage of evolutionary feature selection for isolating context-sensitive cues tone, slang, abusive vocabulary in sparse, high-dimensional datasets.

Our study confirms these results on Arabic Instagram comments, with all classifiers registering precision improvements between 2.92 pp and 4.76 pp. Comparable PSO-based feature selection for Turkish text classification, which yielded precision and accuracy boost, underscoring the value of metaheuristic selection in complex languages^47^.

By comparing our GA-FS with these prior works, we observe consistent gains in accuracy and F1-scores across different scripts and dialects, evidence of our method’s robustness and transferability to other low-resource languages.

### Study limitations

Several limitations warrant consideration:

- Dataset Specificity.

The study was founded on a single corpus of Instagram comments, that may not reflect the full variety of Arabic dialects or platform norms. Generalizability to other social media contexts (e.g., Twitter, Facebook) has yet to be demonstrated. Additionally, mapping cyberbullying solely through sentiment labels introduces potential misclassification, as neutral comments may still carry harmful intent.

Another limitation is that, although the chosen GA settings proved effective in this study, alternative populations and generations may further benefit from more complex feature spaces or datasets; a broader parameter sensitivity analysis could be explored in future work.

Another limitation is that we did not directly compare the GA to other standard feature selection or dimensionality reduction techniques (e.g., Chi-Square, Mutual Information, PCA); therefore, it remains unclear whether GA is optimal versus alternatives for this dataset. While metaheuristic algorithms like GA are often preferred in recent text classification studies because they select features tied to a classification-relevant fitness function, formal benchmarking remains necessary to confirm this advantage empirically.

### Future directions

To extend the present work, several research avenues are recommended:

- Cross-Platform Validation.

Validating the GA approach on corpora spanning various platforms (microblogs vs. photo sharing apps) would ascertain its robustness in various linguistic contexts.

- Real-Time Monitoring Systems.

Implementing the best models in real-time cyberbullying monitoring dashboards could serve as proof of concept for mental health screening, crisis management, or market analysis.

- Explainable AI Techniques.

Combining GA selected features with explainability models (e.g., SHAP, LIME) can also help explain the relationship between retained metrics and cyberbullying detection outcomes, strengthening interpretive findings.

- Comparative Analysis of Feature Selection Standard Methods.

Comparing GA-FS with typical filter and dimension reduction methods so that their properties as well as situations under which each method performs optimally can be known better.

- Parameter Sensitivity Analysis.

Conducting a thorough parameter sensitivity exploration of gene algorithm parameters to determine if alternative configuration can provide added benefits, especially for processing larger or more varied datasets.

- Advancing Contextual Understanding.

Enhancements to the framework should focus on improved contextual analysis, aiming to better detect subtle or ambiguous cases of cyberbullying that simpler sentiment-based methods might overlook.

## Conclusion

In this study, it was demonstrated that GA–FS can halve the dimensionality of an Arabic language cyberbullying detection dataset without compromising and more typically improving classification accuracy and efficiency. The approach refines model decision boundaries, stabilizes ensembles, and accelerates processing, thereby yielding an interpretable and scalable solution to the study of harmful social media expressions in psychological research. Ongoing research with transformer embeddings and validation extension across dialects will further enhance the advancement of language-aware cyberbullying analysis tools to Arabic linguistic sophistication.

### Methods

**Fig. 3:**
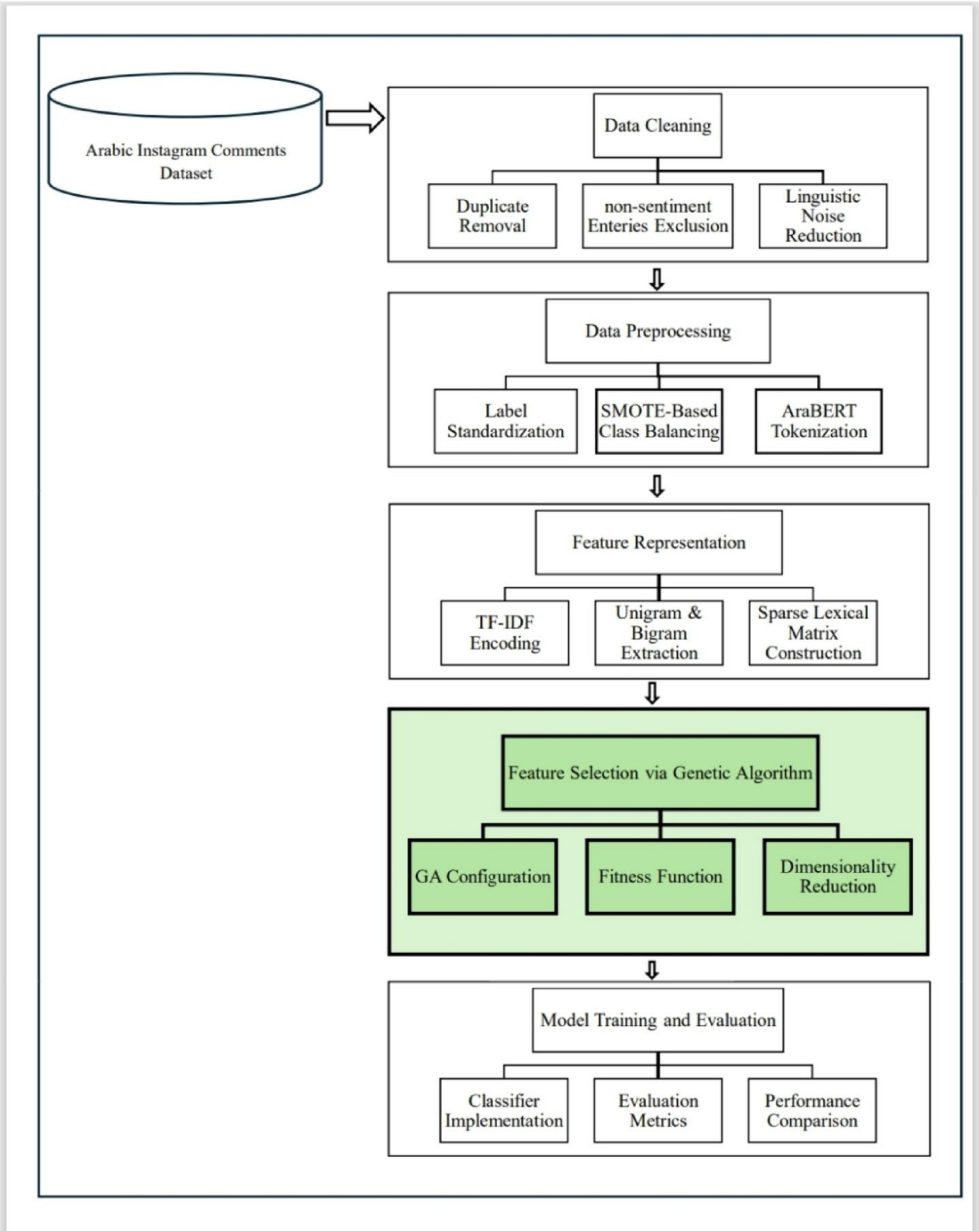
Schematic overview of the pipeline framework for GA-FS-based Arabic cyberbullying detection.

This study introduces a machine learning framework designed to classify Arabic-language Instagram comments for cyberbullying detection, operationalized using three labels positive (not cyberbullying), negative (cyberbullying/toxic), and neutral (uncertain), with an emphasis on improving detection performance through GA -FS. The methodology proceeds in several stages, beginning with dataset preparation and text preprocessing, followed by feature representation and selection, and concluding with model training and evaluation. Each step is described in detail below and illustrated in Fig. 3.

### Dataset and initial cleaning

The dataset used in this study consists of 46,898 user comments written in Arabic and collected from Instagram. Unlike typical sentiment analysis tasks, each entry here was annotated according to cyberbullying detection guidelines with one of three labels: positive (not cyberbullying), negative (cyberbullying/toxic), or neutral (uncertain). Although the data was labeled, the raw dataset, which originated from earlier published work^44^, was not directly suitable for training due to noise and inconsistency. Duplicated entries were identified and removed, and records lacking either a valid cyberbullying label or content were excluded. Minor formatting inconsistencies in the label field (e.g., “positive” vs. “Positive”), were also corrected by standardizing case and spacing.

After applying the cleaning steps, the usable dataset was narrowed down to 39,066 comments. The distribution of cyberbullying detection labels was not balanced, negative entries outnumbered both positive and neutral ones by a clear margin. (Negative: 45.4%, Positive: 44.5%, Neutral: 10.1%). To preserve the original distribution in evaluation, the data was stratified and partitioned into training (80%) and testing (20%) subsets, maintaining class proportions in both.

### Class imbalance mitigation

At that stage, the class imbalance was addressed more directly. The training portion was enhanced.

through the use of Synthetic Minority Over-sampling Technique (SMOTE), where the synthetic data points are generated from the existing ones. The approach avoids simply replicating and instead builds new entries that resemble those of the minority classes. Through the application, each cyberbullying class in the training data concluded with 17,742 records, amounting to 53,226. That balance was important to ensure the classifiers would not be skewed toward the overrepresented class. Although this strategy improved balance across classes, it also introduces certain challenges. While SMOTE can effectively balance the training data, generating synthetic samples in the high-dimensional, sparse TF-IDF feature space may lead to examples that are not fully semantically meaningful; future work should also consider alternative methods such as cost-sensitive learning.

### Text preprocessing

For preparing the textual data in a form suitable for feature extraction and modeling, preprocessing was done. The non-textual and non-informative characters like punctuation marks, digit (Arabic and Latin), emojis, user mention and hyperlinks were removed (https? ://\S+), (@\w+), Elongated letters and repeated characters, commonly used in informal Arabic for emphasis (e.g., “جدااا” → “جدا”), were normalized to their base forms. Additionally, some letters forms such as Alef forms (أ → ا) and Yeh variants (ي ↔ ى) was standardized and a curated list of over 500 Arabic stop words (e.g., “ثم” ، “ليس”) was applied to reduce lexical noise, while key semantic elements such as negation particles and sentiment-shifting terms were retained due to their importance in polarity relevant to toxic language.

Tokenization was carried out using the pre-trained AraBERT v0.2 tokenizer, which was selected for its strong performance in segmenting Modern Standard Arabic as well as informal dialectal structures common to social media content. This tokenizer effectively addressed morphological variations and idiomatic expressions that might otherwise degrade the quality of token boundaries.

### Representation of features

After preprocessing and tokenization, text data were represented as numerical formats by TF-IDF.

This process provides more weightage to words that occur frequently within a document but less frequently in the entire corpus as a whole and hence highlights terms with discriminative semantic content. Unigrams and bigrams were both used to capture single words and frequent combinations of two words. The sparse feature matrix that was obtained contained 38,035 unique dimensions, giving rich lexical representation of the corpus on the other hand resulted in having problems with dimensionality and sparsity.

### Feature selection using GA

To constrict computational efficiency and suppress overfitting, feature selection was completed using GA. This evolutionary algorithm evolves subsets of features iteratively based on the weighted F1-score of a majority class baseline classifier as the fitness function. GA was initialized with a population of 20, evolving to 10 generations. Crossover rate of 0.5 and mutation rate of 0.2 were employed, with tournament selection being used to select high-performance subsets of features to proceed to the next generation. These parameter values were chosen after preliminary experiments with larger population sizes and more generations, which resulted in no substantive improvement in performance but significantly increased computation time; this is consistent with default settings commonly reported in the literature for high-dimensional text features.

Specifically, the fitness of each feature subset (chromosome) was evaluated by training an SVM model and computing its weighted F1-score on a hold-out validation partition. SVM was chosen because it demonstrated strong baseline performance in preliminary experiments and is widely regarded for its robustness and accuracy in high-dimensional text classification settings.

The GA efficiently reduced the feature space from 38,035 to 19,012 dimensions by eliminating redundant and non-informative features. The reduction preserved semantic meaning while improving model interpretability and computational efficiency.

### Model training and evaluation

To assess the leverage of the selected features, four classical machine learning classifiers were utilized: SVM, RF, LR, and MNB. Each model was tested on both the full TF-IDF feature set and the reduced feature set resulted from GA.

- The SVM was implemented using a linear kernel with a regularization parameter of C = 1.0, selected after hyperparameter tuning.
- The RF model consisted of 100 estimators, using default settings for depth and node splitting.
- LR was allowed up to 1000 iterations to ensure convergence, particularly under high-dimensional input.
- MNB was trained using standard configuration parameters due to its proven robustness in text classification tasks.

Each model’s performance was assessed using accuracy, precision, recall, and F1-score, with classification runtime also recorded to evaluate computational efficiency. Findings revealed that GA-FS improved classification performance substantially and reduced execution time systematically. For example, SVM’s F1-score improved from 66.0% to 71.61%, while its runtime reduced from 11.6 s to 5.0 s. Similar improvements in precision and computational efficiency were felt by the other classifiers to be discussed in the results section.

## Data Availability

The data used in the current study is publicly available and was obtained from a previously published source [44]. Full citation details are provided in the References section. The specific preprocessed version employed in this research is available from the corresponding author upon reasonable request.
